# Long‐term monitoring of a population of greater horseshoe bat emphasises the importance of a pest beetle prey on demographic trends

**DOI:** 10.1002/ece3.70323

**Published:** 2024-09-29

**Authors:** Stefano Mammola, Alberto Pastorino, Paolo Debernardi, Elena Patriarca, Laura Garzoli

**Affiliations:** ^1^ Molecular Ecology Group (MEG), Water Research Institute (IRSA) National Research Council (CNR) Verbania (VB) Italy; ^2^ NBFC, National Biodiversity Future Center Palermo Italy; ^3^ Laboratory for Integrative Biodiversity Research (LIBRe), Finnish Museum of Natural History (LUOMUS) University of Helsinki Helsinki Finland; ^4^ Frazione Chaillod 10/4 Saint‐Nicolas Italy; ^5^ S.Te.P. Carmagnola (TO) Italy

**Keywords:** birth timing, cockchafer, Maybug, *Melolontha*, predator–prey interactions, *Rhinolophus ferrumequinum*

## Abstract

The global decline in insect biomass has far‐reaching implications for terrestrial and freshwater food webs, impacting species reliant on insects as a crucial component of their diet. This issue extends to species traditionally considered agricultural pests, such as the common cockchafer *Melolontha melolontha*. In the race to combat cockchafers through collection, insecticide use, and other control methods, the repercussions of their numerical fluctuations on predators, including species of high conservation importance like bats, have been largely overlooked. Drawing on 31‐years of monitoring data for a greater horseshoe bat *Rhinolophus ferrumequinum* population in the Aosta Valley (Western Italian Alps), we investigated whether annual fluctuations in bat counts are influenced by cockchafer availability and weather conditions. Despite an overall positive trend in bat abundance, we observed pronounced annual fluctuations, mostly driven by cockchafer availability rather than variations in temperature and precipitation. Furthermore, we found a significant association between cockchafer availability and the median date of birth and birth rate of bats. Births occurred approximately 5 days earlier in cockchafer flight years, with earlier births also linked to warmer spring temperatures and higher numbers of warm days in April. Moreover, the ratio pups/older bats was 0.56 in cockchafer flight years, compared to 0.47 in other years. Our results underscore the importance of considering predator–prey dynamics when examining the long‐term population trends of species of conservation concern. We recommend implementing restrictions on the use of chemicals and other potentially harmful practices that may diminish prey abundance or quality, including that of species considered as agricultural pests. In designing conservation strategies, a delicate balance should be struck between the current interests of farmers and the overarching goal of preserving biodiversity against potential future threats.

## Graphical abstract

We present the results of 31 years of monitoring data for a greater horseshoe bat (*Rhinolophus ferrumequinum*) population in NW Italy. We investigated whether annual fluctuations in bat counts are influenced by common cockchafer availability and weather conditions. Our results underscore the importance of considering predator–prey dynamics when examining the long‐term population trends of species of conservation concern.

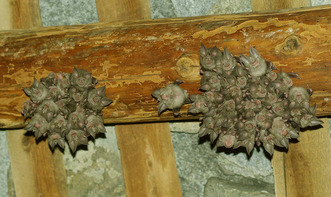

## 1 INTRODUCTION

In recent years, evidence has accumulated about an alarming decline of insect populations across the globe (Cardoso et al., 2020; Wagner et al., 2021). This phenomenon, often referred to in the media as the “insect apocalypse” (Cardoso & Leather, 2019), was first highlighted in a study published in 2017, which found that the biomass of flying insects in German nature reserves had declined by more than 75% over just 27 years (Hallmann et al., 2017). Since then, other studies have confirmed insect population declines across many areas of the world and how previously common species are becoming harder and harder to find (e.g., Powney et al., 2019; Seibold et al., 2019; van Klink et al., 2023). The causes for these declines are diverse, often involving a combination of factors, such as habitat loss and degradation, widespread use of polluting and harmful substances, spread of non‐native invasive species, global climate change, and direct overexploitation (Cardoso et al., 2020). With the disappearance of insects, we are losing key nodes of terrestrial and freshwater food webs. Inevitably, these losses cause ripple effects on other ecosystem components, especially on species that feed on insects. Yet, public awareness of the problem is still scarce and concern is generally limited to a few pollinator species, notably bees. In this context, stating that the decrease of insect pest species can also have negative ecological consequences may look far too radical. Nevertheless, the problem actually exists and it should be taken into account for the purpose of a correct land management (Samways et al., 2020).

The case of the common cockchafer *Melolontha melolontha* (Linnaeus, 1758), considered one of the worst pests for agriculture, is emblematic. The adults of this beetle emerge from mid‐April to mid‐June (with a peak in May, hence the common name Maybug) and feed on the leaves and flowers of many deciduous trees, including economically important species such as walnut and apple trees. Damages are mainly caused by the larvae, which take a long time to develop, living in the soil and feeding on the roots of a large variety of plants (CABI Compendium, 2022; Huiting et al., 2006). For centuries, attempts to control cockchafers have involved collecting and killing adult beetles. In the second half of the 20th century, the use of chemicals like DDT and lindane, combined with the environmental transformations caused by the mechanisation and intensification of agriculture, brought the common cockchafer close to extinction in large areas of Europe (Robert et al., 1986; Sellier & Madrolles, 2023; Zimmermann, 2010). Subsequently, highly toxic pesticides were banned, and currently, in some areas, cockchafer populations are recovering (Wagenhoff et al., 2014; Woreta, 2015). Today, eradication efforts against this beetle primarily continue by biological methods (introducing pathogenic fungi or nematodes to kill the larvae), using light traps, pheromones, and other chemical attractants for massive trapping of the adults, or employing mechanical means, such as covering the soil with nets to prevent the swarming of adults (Huiting et al., 2006; Malusá et al., 2020; Trdan et al., 2019; Woreta, 2015).

In this race to control cockchafers, the consequences of their numerical fluctuations on their predators are overlooked, even when species of conservation concern are affected, such as bats. Cockchafers, weighing about 1 g and having a thin chitinous skeleton, are highly nutritious, making them a profitable prey for some large bat species (Hoese & Schneider, 1988). Moreover, their seasonal availability coincides with bat pregnancy, a period when food resources are crucial. In temperate zones, the length of bat pregnancies is influenced by the fact that bats can alternate periods of activity and successful foraging with periods spent in torpor due to unfavourable environmental conditions (bad weather and scarcity of prey); while in torpor, foetal development slows down, resulting in a longer pregnancy and late parturition (Racey & Entwistle, 2000). A study conducted in SW‐Switzerland demonstrated that in years when cockchafers are abundant, females of the lesser mouse‐eared bat *Myotis blythii* (Tomes, 1857) give birth, on average, 10 days earlier than in years without cockchafers (Arlettaz et al., 2001).

Common cockchafers are also preyed upon by the greater horseshoe bat *Rhinolophus ferrumequinum* (Schreber, 1774). Recent studies based on DNA metabarcoding of faecal samples have shown that this bat can be considered a generalist predator, as it consumes a wide range of taxa (Alberdi et al., 2020; Tournayre et al., 2021); nevertheless, some prey items occur in its diet far more frequently than others (Tournayre et al., 2021). Unfortunately, these two studies are based on samplings carried out from June to October, that is, in months of scarce or no availability of cockchafers. Moreover, they possibly include samplings performed in years when cockchafer availability was low because of the beetle life cycle, or conducted at sites where cockchafers are anyway rare or absent. Previous research, which used less sensitive morphology‐based microscopy analyses but included spring samples collected in presence of cockchafers, proved that these beetles are largely consumed by *R. ferrumequinum* (Boireau & Le Jeune, 2007; Flanders & Jones, 2009; Ransome, 2020; Roué & Barataud, 1999), and that, as long as cockchafers are abundant, they can be the preferred prey for this bat (Ransome, 1996; Ransome & Priddis, 2005).

Long‐term studies on English and Welsh populations of *R. ferrumequinum* have demonstrated that early parturition dates in this species favour the survival of both young bats and their mothers during the following spring (Ransome, 1989, 1995). Late‐born cohorts of individuals have a lower growth rate (McOwat & Andrews, 1995) and die out in a few years, unlike the early‐born ones, which provide the bulk of the colony's offspring (Ransome, 1989; Ransome & Hutson, 2000). Common cockchafers have been suggested to be a prey item that probably promotes rapid pregnancy when present (Ransome & Hutson, 2000). Moreover, a long‐term study of one maternity colony has highlighted a significant positive relationship between the time of beetle appearance in the diet of the bats and the mean birth date of the same year (Jones et al., 2015).

*Rhinolophus ferrumequinum* is currently considered as Least Concern on the International Union for Conservation of Nature (IUCN) Red List, but it underwent dramatic declines in Europe during the second half of the last century, which took it to extinction or close to extinction in several countries (Piraccini, 2016). Reasons for this decline included the use of highly toxic pesticides in agriculture and forestry and of timber preservative in roosts, habitat loss and fragmentation, reduction of food availability, and roost disturbance (Barova & Streit, 2018). In Italy, the species is still classified as Vulnerable (Rondinini et al., 2022).

We monitored a population of *R. ferrumequinum* in the Aosta Valley (NW Italian Alps) by regularly surveying a hibernaculum and a maternity colony. In contrast to other areas of Northern Italy, this is a region where cockchafers still occur (Pedrazzini et al., 2023). When abundant, they appear to be strongly preyed upon by greater horseshoe bats, as suggested by the presence of their remains inside the maternity site and beneath feeding perches. Our long‐term survey, covering a 31‐year period, enabled us to establish the trend of the local bat population and test the prediction that local population fluctuations and demographic parameters should be driven by cockchafer availability and weather conditions. We investigated the role of weather variables in conjunction with prey availability since spring weather has been identified as a factor conditioning birth timing and population trends of *R. ferrumequinum* in other areas (Andrews et al., 2022; Froidevaux et al., 2017; Ransome & McOwat, 1994) and autumn weather is known to contribute to juvenile mortality before hibernation starts (Ransome, 2020). Weather also affects insect availability (Ransome, 1997; Taylor, 1963), but this applies only marginally to cockchafers, whose adult swarms peak every 3 or 4 years depending on the geographic area, regardless of local climatic conditions (Hurpin, 1962). Referring to such years as “flight years”, we made these explicit predictions: (1) positive bat population fluctuations in the maternity colony should occur preferentially when the spring of the previous year corresponded to a flight year and was characterised by good weather. Such conditions favour foraging and provide high quality prey; we therefore expected that, by promoting gestation (see below), they should enhance the survival of females and their offspring, resulting in an increase in the number counted the following year; (2) positive bat population fluctuations at the hibernaculum should occur preferentially when the previous autumn and spring were characterised by good weather and corresponded to a flight year. We expected that these conditions should enhance the survival of females and their offspring born in the year, and diminish juvenile mortality before hibernation; (3) births should occur earlier in flight years, as well as in years with warmer, less rainy springs, since these conditions reduce the need for pregnant females to spend time in torpor; (4) the proportion of pups to older bats in the maternity colony should be higher in flight years and in years with favourable weather, due to a lower abortion rate among pregnant females.

Finally, to verify the regularity of the cycle in the Aosta Valley and to gather information on local differences in cockchafer abundance over time, we examined documents preserved in historical archives.

## 2 MATERIALS AND METHODS

### 2.1 Bat counts

The Aosta Valley is the northwesternmost region of Italy. A description of the area and its bats is provided in the regional bat distribution atlas (Patriarca & Debernardi, 2021). In Aosta valley, the only hibernaculum known to be used by a large number of greater horseshoe bats is the mine complex included in the Special Area of Conservation (S.A.C.) IT1205034 *Castello e miniere abbandonate di Aymavilles*, part of the Natura 2000 network of protected areas. These mines were abandoned in 1976, and during the winter 1992/93 it was identified that they were used by bats (Baratti et al., 1994). Since then, we carried out winter counts of the individuals that hibernate in the site, operating once a year (mainly in January) to limit disturbance (Battersby, 2010).

In 2000, at a distance of 5.8 km from the mines, we discovered the only maternity colony of greater horseshoe bats of Aosta Valley. The colony roosts inside the garrets of the Cathedral of Aosta. According to local witnesses, bats have been seasonally using the building for at least 50 years, and the colony was once much larger than it is today. Among the factors that may have affected colony size is the disturbance caused by renovation works made towards the end of the last century.

After its discovery, the colony has been included into the same S.A.C. of the mines (IT1205034), ensuring its protection, and yearly monitored. We counted the individuals aged ≥1 year (hereafter generically called “adult bats”, although also including subadult individuals) from videotapes recorded during bat emergence at dusk. Over the years, we took videos using either thermal cameras or highly sensitive video cameras operating in infrared light. We used two cameras during each survey, one for each window used by the bats to exit the building. When the emergence ended we entered the roost, where (if present) we counted the few adult bats that had remained inside and photographed the pups. We counted the number of pups from the photos and by direct observation. Referring to the descriptions provided by Ransome (1998 and 2020), we estimated when pups were born based on their morphology (approximative size, absence/presence of ventral fur, eyes closed/open) and behaviour (hanged alone or in groups, capable of wing flapping, capable of flight). We estimated the median birth date of each year as the date when at least half of the pups counted were born.

In the first 11 years of censuses (2001–2011), we carried out three counts each year: at the end of June, in the middle of July, and at the end of July/beginning of August. Having verified that no birth occurred after the 20th of July and that in the third count it was impossible to distinguish adult from young bats from the video footage, starting from 2012 we limited the yearly censuses to the first two counts. For the purpose of this work, for each year, we took into consideration the highest number of adult bats counted in one census and the number of pups resulting from the counts after having excluded all the possible double counts. We used the ratio pups/adults as a proxy for the birth rate.

### 2.2 Weather variables

We obtained time series of mean temperatures and total precipitation data with daily resolution from the meteorological station of Saint Christophe – Aeroporto, situated at a linear distance of 2.9 and 8.1 km from the Aosta Cathedral and the hibernaculum, respectively. The station is managed by Regione Autonoma Valle d'Aosta (Centro Funzionale Saint‐Christophe, https://cf.regione.vda.it/portale_dati.php).

### 2.3 Common cockchafer availability

We assembled data about current and past availability of cockchafers in the Aosta Valley through a literature survey and by consulting historical sources at several libraries and archives: the Biblioteca Nazionale di Torino, Biblioteca digitale valdostana (http://cordela.regione.vda.it/), Archivio Storico Regione Autonoma Valle d'Aosta, Biblioteca del Settore Fitosanitario della Regione Piemonte, and the digital archives of the municipalities of the Aosta Valley. We also checked if standardised data on cockchafer abundance were available by consulting the regional offices (Servizio Fitosanitario Regione Autonoma Valle d'Aosta). Standardised censuses of larvae were conducted from 1997 to 2003, but no data on adults was available.

Based on the overall information collected, it was possible to identify series of “flight years” across time. Given these data, each year was then classified as a binary variable, “Cockchafer flight: yes/no”.

### 2.4 Statistical analyses

We conducted analyses in R version 4.1.0 (R Core Team, 2021). We ran multiple regression models (linear models and generalised linear models; see below) to test our working hypotheses. In all the analyses, we followed the general approach outlined by Zuur and Ieno (2016) for data exploration, model fitting, and validation.

Prior to model fitting, we explored the dataset, visually inspecting variable distribution, the presence of outliers, and collinearity among continuous predictors (using pairwise Pearson's correlations, setting a threshold for collinearity at Pearson's *r* > ±.7).

**FIGURE 1:**
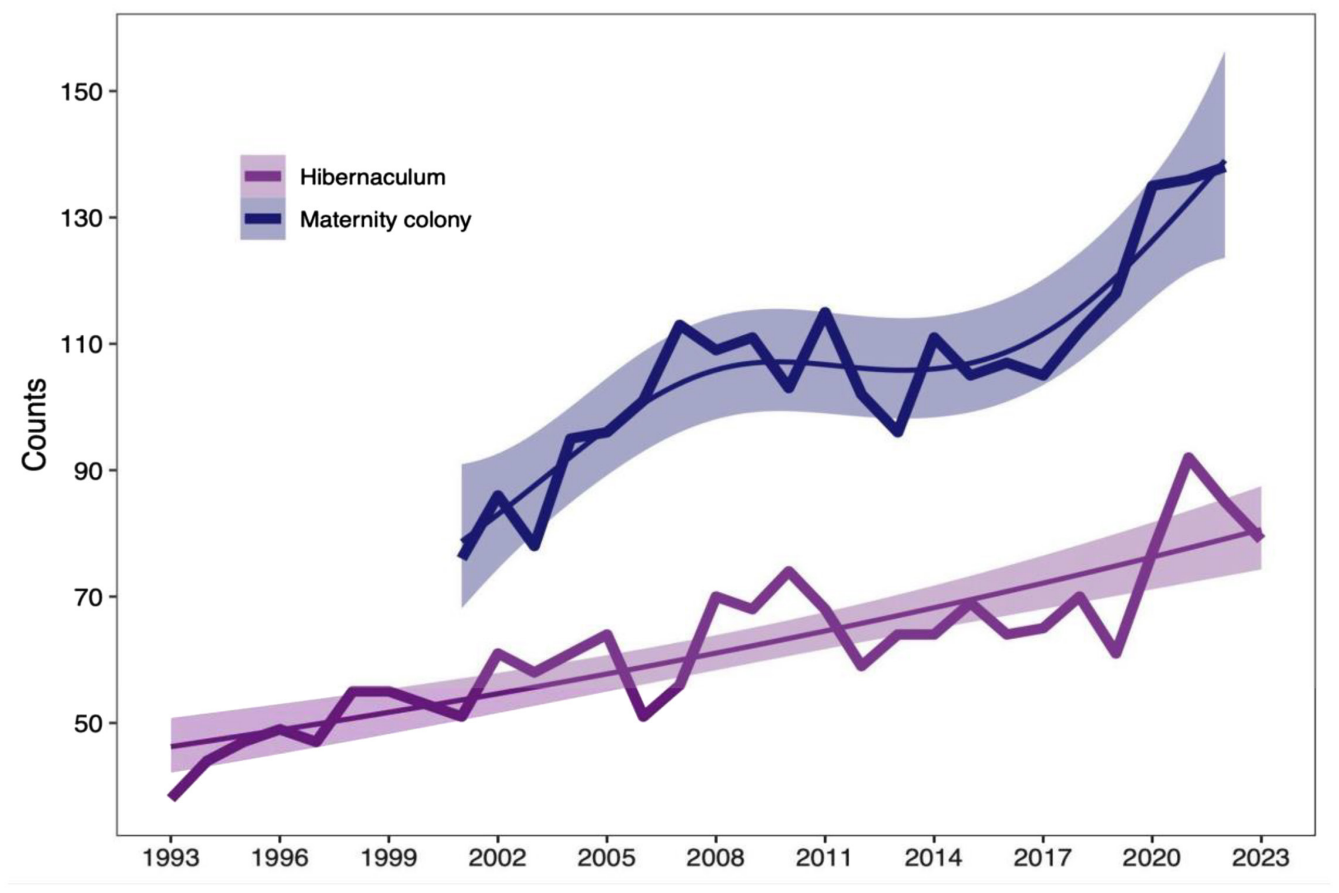
Population trends based on the numbers of adult bats in the maternity colony and the numbers of individuals in the hibernaculum. Broken thick line is the observed data, the smoothed thin lines are the predicted population trend based on Poisson generalised additive models fitted to the data, and the shaded grey areas are the 95% confidence intervals associated with each prediction.

In the first set of models, we explored the role of weather and cockchafer flight (namely, whether a given year was a flight year) in determining the population trend in the maternity colony. To this end, we first fitted a Poisson generalised additive model with the R package ‘gam’ (Hastie, 2020), predicting the relationship between the number of adult individuals and year of census (Figure 1). For each year, we then extracted the residuals from the regression curve, whereby positive residuals indicate that the population trend is greater than the expected given the overall population trend across the monitored period, and negative residuals vice versa. Next, we fitted three linear models to test the relationships between the population trend (i.e., expressed as residuals) and three sets of weather variables (mean temperatures and total precipitation of previous year spring, previous autumn, and previous winter, respectively) and cockchafer flight year. Here and below, we assumed the spring period from 15 April to 15 June (in order to represent the approximately two months gestation period, since at the studied site births occur from the middle of June onwards), as autumn the months of September and October (important for fat storing before hibernation), and as winter the trimester December–February (core of the hibernation period). Note that we log‐transformed the variable total autumn precipitation to minimise the influence of one outlier. Note also that we constructed separate models for the three sets of weather variables due to the reduced sample size and potential collinearity among them. For the same reason, we did not consider interactions among variables.

In the second set of models, we explored the role of weather and cockchafer flight in determining the population trend at the hibernation site. To allow comparability with the first set of models, excluding possible bias due to disturbance factors that might have affected the maternity colony before it was discovered, we analysed the data starting from the winter 2001/02. As in the previous case, we extracted the residuals from the population trend modelled through a generalised additive model (Figure 1). We fitted two linear models to test the relationships between the population trend (residuals) and two sets of weather variables (mean temperatures and total precipitation of previous spring and previous autumn, respectively) and cockchafer flight (whether the previous spring occurred in a flight year). Note that in the model including weather variables for autumn, precipitation and temperature were collinear, and thus we only included the temperature variable in the model.

Next, we tested if weather variables and cockchafer flight influence birth timing. Given that the mean date of birth is a positive integer, we fitted a generalised linear model assuming a Poisson error (i.e., a distribution suitable for count data). In the first model, we tested the relationships between the median date of birth and the total precipitation and mean spring temperature, as well as cockchafer flight. In the second model, we included, as a weather variable, the count of April days with a mean temperature above 10°C. We did not perform the same analysis on May data, since in that month almost all days had a mean temperature above 10°C. According to literature, this temperature value is a threshold above which the activity of many insects strongly increases (Jones et al., 1995; Rydell, 1989); moreover, April temperatures have been recognised as a crucial factor affecting birth‐timing in other areas (Andrews et al., 2022; McOwat & Andrews, 1995).

Finally, we tested if weather variables and cockchafer flight have an influence on the birth rate, estimated by the proportion of juveniles to adult individuals in the maternity colony. We modelled the proportion with a Binomial generalised linear model, including spring mean temperature, total precipitation, and cockchafer flight as covariates.

We validated all models with the function *check_model* from the R package ‘performance’ version 0.0.0.6 (Lüdecke et al., 2020). This function generates standard diagnostic plots based on residuals and fitted values, including a visualisation of normality of residuals, the degree of multicollinearity in the covariates included in the models (based on variance inflation factors), and an assessment of potential outlying observations. For Poisson and Binomial models, we also checked overdispersion with the function *check_overdispersion* from ‘performance’. Taking into account the reduced sample size, all diagnostic checks indicated the models to be satisfactory.

## 3 RESULTS

### 3.1 Historical notes on common cockchafer availability

According to the literature on the attempts to control common cockchafers carried out in the Aosta Valley from the 1980s (Bondaz, 1996, 1997; Bondaz et al., 2002; Guglielmo et al., 2007) and information provided by the Servizio Fitosanitario Regione Autonoma Valle d'Aosta (Bonfanti R., pers. com.), this beetle appears in the region in a cycle of 3 years; the last flight year occurred in 2022. In older sources (*Bulletin de la Comice Agricole d'Aoste* 1869–1921; *Almanach de l'agriculteur valdôtain*, 1885; *Archivio storico del Comune di Aosta*, 1923–1932; Fusinaz, 1926) we found evidence that the years 1872, 1875, 1878, 1881, 1884, 1911, 1917, 1923, 1929, 1932, 1935, 1950, 1962, and 1965 were also massive flight years, suggesting that the cycle has occurred regularly since at least 1872. Remarkably, Henry (1929) mentions a religious procession from Aosta to Morgex, aimed to eradicate cockchafers, which took place in 1644, a year that is also consistent with this time series. It is believed that the common cockchafer, in addition to the short‐term cycle mentioned above, undergoes a longer cycle of about 30–40 years, culminating in peaks of exceptionally high numbers (Billamboz, 2014; Huiting et al., 2006; Robert et al., 1986); however, we could not find any data from which to identify with certainty the years of such cycle in the Aosta Valley.

In the past, the attempts to reduce cockchafer numbers in the region were performed by manual collection of the adults during their swarming period in the peak years of the cycle. Such activity was initially organised on a voluntary basis, but in 1913 a national law (L. 26 June 1913 n. 888) established that it could be made mandatory by the prefectures, which indeed happened in the Aosta Valley starting from 1923 (Fusinaz, 1926). Collectors were rewarded based on the quantity of cockchafers removed, and the weight of beetles destroyed was recorded in public registers. Some of these documents were unfortunately destroyed; consequently, we found only partial data in the archives we consulted. These data suggest nonetheless that cockchafers were once far more abundant than today, since in some years (for example in 1923, 1932 and 1935) up to 25–30 millions of individuals were collected. Manual collection continued, although inconsistently, during the 20th century. The last collection occurred in 1986, and brought to the destruction of about 1.5 millions of individuals (Bondaz, 1997). In the 1960s, chemical treatments with organophosphate pesticides were applied by aerial dispersion (from helicopter) to fight cockchafers in the region (Bondaz et al., 2002). In the 1990s, the use of an entomopathogenic fungus (*Beauveria brongniartii* (Sacc.) Petch) was tested as a control agent against the beetle (Bondaz & Vallet, 1996; Cravanzola et al., 1996). Having found that this method gave scarce results due to local environmental conditions, a more virulent strain of the agent was selected to be used (Dolci et al., 2006). In the last years, plastic nets have been deployed in apple orchards to prevent egg laying in the soil by cockchafer females during flight years, a method that had already proved to be effective in protecting orchards and reducing grub density in Trentino‐Alto Adige (Varner & Mattedi, 1996; Zelger, 1996) and Switzerland (Brenner & Keller, 1996). Additionally, one treatment with Acetamiprid (a neonicotinoid insecticide) was permitted during the swarming of adult cockchafers, even though it was not commonly used. Together with the abovementioned actions, the environmental transformations that have occurred in the valley bottom from the mid‐20th century must have played a role in reducing cockchafer abundance: the surface occupied by tree‐lined meadows and pastures has been strongly reduced, while built area and artificial night lighting have increased.

### 3.2 Size, trend, and factors affecting greater horseshoe bat population

Abundance of bats in both the maternity colony and hibernation site showed positive trends over the monitored period (Figure 1). Specifically, the number of adult bats in the maternity colony significantly increased from 76 to 138 individuals between 2001 and 2022 (Poisson generalised additive model: sum of squares: 35.936, *F* = 119.79, *p* = 4.059 e^−09^; *R* ^2^ = .973). This represents an overall percentage increase of 81.58%, although there was a period of relative stasis between 2007 and 2015.

The number of individuals at the hibernation site varied between 38 and 92, significantly and steadily increasing between the winter 1992/93 and the winter 2022/23 (Poisson generalised additive model: sum of squares: 46.693, *F* = 103.20, *p* = 2.331 e^−10^; *R* ^2^ = .942). This represents an overall percentage increase of 142.11%. There was a high correlation between the number of individuals in the maternity colony and in the hibernation site (Pearson's *r* = .765; correlation test: *t* = 5.325, df = 20, *p* = 3.265 e^−0.5^).

**FIGURE 2:**
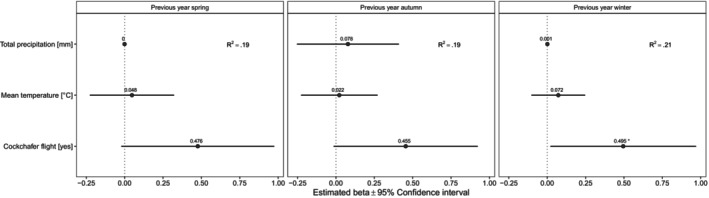
Factors influencing the population trend of adult bats in the maternity colony. Forest plots summarise the estimated parameters based on linear models. The three models separately test alternative relationships depending on cockchafer cycle and weather conditions. Error bars mark 95% confidence intervals. Variance explained is reported as *R* ^2^. Asterisks (*) mark significant effects (*α* = .05).

Population trend in the maternity colony was affected by cockchafer availability, whereby the population trend was positive if the previous year was a flight year. This effect was positive and significant (or close to significant) in all models (Figure 2). Conversely, none of the weather variables significantly affected population trends (Figure 2).

**FIGURE 3:**
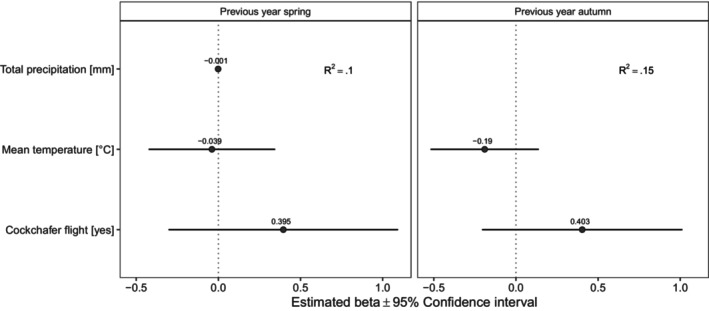
Factors influencing the population trend at the hibernaculum. Forest plots summarise the estimated parameters based on linear models. The two models test alternative relationships considering only weather conditions in the previous spring and previous autumn. Error bars mark 95% confidence intervals. Variance explained is reported as *R* ^2^. Asterisks (*) mark significant effects (*α* = .05).

Regarding the hibernating bats, we found no significant relationship with any of the variables considered, although cockchafer flight approached statistical significance and its effect was in the expected positive direction (Figure 3).

**FIGURE 4:**
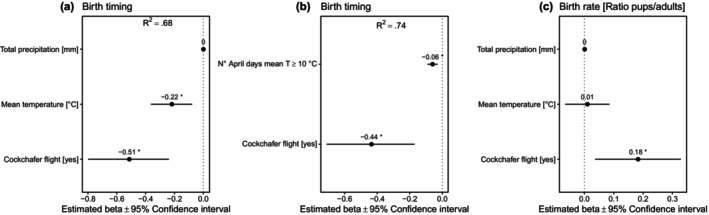
Factors influencing birth timing, expressed as median birth date (a, b), and birth rate, estimated as the ratio pups/adults (c). Forest plots summarise the estimated parameters based on Poisson (a, b) and Binomial (c) generalised linear models. Error bars mark 95% confidence intervals. Variance explained is reported as *R* ^2^. Asterisks (*) mark significant effects (*α* = .05).

Birth dates varied within the period from 14 June to 20 July; the most frequent birth date was 2nd of July. The median date of birth was significantly associated with cockchafer availability, namely births occurred about 5 days earlier in flight years (Figure 4a,b). Furthermore, births occurred earlier when the spring mean temperature was warmer (Figure 4a) and in years when the number of warm days in April was higher (Figure 4b). The birth rate was significantly affected by cockchafer availability (mean birth rate [±SD] of .56 ± .11 in cockchafer flight years – ratio pups/adults, e.g., 56 pups per 100 adults – versus .47 ± .07 in the other years), but not by weather variables (Figure 4c).

## 4 DISCUSSION

### 4.1 Drivers of long‐term population trends

Our study demonstrates that the population of *R. ferrumequinum* of the Aosta Valley has increased during the last three decades. This may appear to suggest that the species is flourishing; however, if anecdotal statements about a past larger size of the maternity colony in the Aosta Cathedral are true, the positive trend observed should be considered simply as a recovery. Moreover, the current size of the maternity colony is smaller than 200 individuals—the number recommended in the Action Plan for this species to guarantee to colonies the viability needed to cope with stochastic demographic fluctuations or random negative events, such as repeatedly adverse climatic conditions (Ransome & Hutson, 2000). Finally, it should be considered that the colony of the Aosta Cathedral has distinctive genetic features (possibly due to isolation and/or to the presence of genetic components from abroad) that make its strict protection especially important (Palladini et al., 2019).

Although we did not mark the bats, the high correlation between the data recorded in the maternity colony and the hibernaculum suggests that the two sites share a good portion of the individuals we counted, hinting that the mines are used by many bats born in the cathedral. The distance between the two sites is within the range (12 km) reported for “type 1” and “type 2” hibernacula, i.e., sites that mainly contain first‐year bats and older immature bats (Ransome, 2020). The lower numbers counted at the hibernation site, compared to those of the cathedral, can be explained in terms of normal bat dispersal.

Long‐term studies on English and Welsh populations of *R. ferrumequinum* proved that spring weather is associated with population trends (Froidevaux et al., 2017) and birth timing. In particular, warm springs are followed by earlier births, with April temperatures playing a key role (Andrews et al., 2022; McOwat & Andrews, 1995; Ransome & McOwat, 1994) especially by conditioning foraging time (Andrews et al., 2022) and prey availability (Jones et al., 2015). During our survey, most births of *R. ferrumequinum* in the Aosta Valley occurred at the beginning of July, later than reported for Western Iran (in late May: Eghbali & Sharifi, 2019) and Bulgaria (in the first 3 weeks of June: Dietz et al., 2006), and slightly earlier than observed in Britain (8–15 July; Andrews et al., 2022; Jones et al., 2015; Ransome & McOwat, 1994). Our results confirm the role of spring and April temperatures in affecting birth timing. They also indicate that cockchafer availability is an even more important factor in anticipating births, and that it conditions the birth rate as well, presumably by reducing the incidence of abortions. Moreover, cockchafer availability affected positively the demographic trend we observed in the maternity colony, suggesting that the earlier birth dates due to this factor may increase the survival chances of young bats and their mothers, as reported in British studies (Ransome, 1989, 1995). A tendency to show a positive correlation with cockchafer flights was observed also in the trend of hibernating bats, although in that case data only approached statistical significance. A lower influence of cockchafer availability on the number of bats counted in the hibernaculum, compared to the maternity colony, could be due to a different sex ratio. In the maternity colony, the proportion of female bats is presumably higher, and cockchafers, occurring during pregnancy, could be more important for female than for male bats.

It is surprising that a prey species currently available only in some years and for a brief period should have an influence on the demography of a predator species. This leads us to wonder what might have happened in the past, when cockchafers were far more abundant than today, as suggested also by the historical data for the Aosta Valley. Nowadays, cockchafers can be considered virtually absent during non‐flight years (Servizio Fitosanitario Regione Autonoma Valle d'Aosta, Bonfanti R., pers. com.), as also described for Switzerland (Arlettaz et al., 2001; Christe et al., 2003). In the past, however, cockchafers must have been present in low numbers also in “normal” years. For example, Della Beffa (1961) wrote that the individuals were far fewer (but still present) in the intermediate years of their cycle in Italy; moreover, data collected in other geographic areas suggest that intermediate years in the past were sometimes characterised by a greater abundance of cockchafers than that observed more recently in the peak years (Cate, 2002).

Arlettaz et al. (2001) suggested that the reduction of cockchafer population observed in many European countries over the past decades may have had strong effects on *M. blythii*, possibly affecting the distribution and population survival of the species. Similarly, negative consequences of cockchafer decrease may be hypothesised for the other bat species that prey upon this beetle, such as the greater mouse‐eared bat *M. myotis* (Arlettaz et al., 2017), the Geoffroy's bat *M. emarginatus* (Vallejo et al., 2023), the Leisler's noctule *Nyctalus leisleri* (Boston et al., 2023), the common noctule *N. noctula* (Lindecke et al., 2020), and the common serotine *Eptesicus serotinus* (Beck et al., 2006; Catto et al., 1996; Kervyn & Libois, 2008). Cockchafer decrease may also affect some birds like shrikes (Cramp & Perrins, 1993) threatened at national (Rondinini et al., 2022) or European level (Birdlife International, 2021, 2022; Burfield et al., 2023): for example, the red‐backed shrike *Lanius collurio* (present also in Aosta valley; Maffei et al., 2018), the southern grey shrike *L. meridionalis* (Lepley et al., 2004), and the lesser grey shrike *L. minor* (Hoi et al., 2004; Krištín & Zilinec, 1998; Lepley et al., 2004). Other bird species linked to extensive agricultural landscape, preying upon cockchafer and present in the study area, are the little owl *Athene noctua*, the Eurasian scops‐owl *Otus scops* (Cramp, 1985), and the Eurasian hoopoe (*Upupa epops*; Arlettaz et al., 2010; Nuhlíčková et al., 2016), whose decline in Switzerland was linked to the decrease of larvae of different insects, among which cockchafer (Fournier & Arlettaz, 2001). In Europe, some falcons (e.g., the red footed *Falco vespertinus* and the hobby *F. subbuteo*) and the European roller *Coracias garrulus* prey on large beetles. Predators feeding on adult cockchafers or larvae include also more common bird species (Cramp, 1985), wild boars, badgers, foxes, many small mammals, and ground beetles (Carabidae; CABI Compendium, 2022).

How is climate change going to affect cockchafers and their predators? Some authors have suggested that climate change is fostering an increase in cockchafer populations (Hann et al., 2008). On the other hand, it is also known that, while rainy and cold weather during cockchafer flights shortens the life span of adult beetles and slows the maturation of the eggs in their bodies, L1 larvae are affected by extreme high temperatures and drought (Huiting et al., 2006). The recovery of some populations of *R. ferrumequinum* has been associated with warmer climate conditions and an earlier peak appearance of cockchafers (Froidevaux et al., 2017; Jones et al., 2015). However, there is also the risk of a future mismatching between the availability of the beetle and the gestation of the bat: due to the increase in temperatures, the life cycle of common cockchafer is expected to accelerate, potentially completing in 2 years and causing adults to emerge in autumn instead of spring. Cases of a shortened life cycle have recently been reported for Austria and Switzerland, including observations of adults emerging in September (Frühwirth, 2020; Pedrazzini et al., 2023). Lastly, climate change might promote cockchafer abundance while negatively affecting alternative key prey species of *R. ferrumequinum*, making cockchafers even more important than today for the conservation of the bat.

### 4.2 Limits of the analysis

We did not include land‐use variables in our analyses, despite their importance in driving long‐term population trends of various bat species. The preferred foraging habitat of *R. ferrumequinum* consists in a mixture of grassland (especially grazed pastures), orchards, and broadleaf woods interconnected with hedgerows and tree lines, whose availability within 3 km from maternity roost influences colony size (Froidevaux et al., 2017). Nevertheless, based on a preliminary inspection of orthophotographs of Aosta Valley (https://geoportale.regione.vda.it/), such landscape characteristics within a radius of 3 km from the maternity site and the hibernaculum did not show noteworthy changes in the last three decades (radical changes occurred between the 50s and 70s).

Changes have taken place instead in the amount of artificial light at night. Sánchez de Miguel et al. (2021), who examined the power of global light emissions detectable by satellites from 1992 to 2017, reported data also for the Aosta Valley, where Artificial Lighting at Night (ALAN) markedly increased along the period. *Rhinolophus ferrumequinum* exhibits a light‐averse behaviour (Rowse et al., 2016), so we can expect that if this factor has any consequence on the demography of the bat, this must be negative, as observed in Great Britain (Froidevaux et al., 2017). Moreover, ALAN could mask the positive effects of cockchafer flight years: lamps, to which cockchafers are strongly attracted (Huiting et al., 2006), could negatively affect the number of these beetles causing their direct mortality and by distracting them from mating and oviposition. At the same time, lamps reduce the availability of cockchafers for *R. ferrumequinum* by concentrating the prey in the light cone, from which the predator is repulsed. Since we had no high resolution data on ALAN, we did not include the variable in the models; nevertheless, we can exclude that ALAN may act positively on the population of *R. ferrumequinum* of Aosta Valley. On the contrary, it could have masked the expected positive effects of other variables; if, notwithstanding this, significant effects are found, these should be under‐ and not overestimated, supporting their important role.

### 4.3 Conservation implications

Given the many factors involved, the conservation of *R. ferrumequinum* in the Aosta Valley requires great caution. The entrance of visitors in the hibernaculum is forbidden, but mounting grilles (permeable to bats) across some of the entrances is needed in order to prevent illegal intrusions and disturbances (Meierhofer et al., 2024; Mitchell‐Jones et al., 2007). Following the recommendations of the Action Plans (Barova & Streit, 2018; Hutson et al., 2001; Ransome & Hutson, 2000), as well as those reported in the Atlas of Bats of the Aosta Valley (Patriarca & Debernardi, 2021) and in a recent project aimed at establishing a Regional Ecological Network (Pastorino et al., 2022), the environmental features that favour foraging and commuting around and between the species' roost sites should be preserved (resulting in wide biodiversity benefits: see Froidevaux et al., 2019). Furthermore, restrictions should be applied to the use of pesticides and other potentially damaging practices which may seriously reduce prey abundance or quality, cockchafer availability included. A balance should be struck between the current interests of farmers and the goal to preserve biodiversity and resilience to possible future threats.

## AUTHOR CONTRIBUTIONS

**Stefano Mammola:** Conceptualization (equal); data curation (equal); formal analysis (lead); writing – original draft (equal). **Alberto Pastorino:** Investigation (equal); writing – original draft (equal). **Paolo Debernardi:** Conceptualization (equal); data curation (equal); investigation (equal); resources (lead); writing – original draft (supporting). **Elena Patriarca:** Conceptualization (lead); funding acquisition (lead); investigation (lead); writing – original draft (lead). **Laura Garzoli:** Conceptualization (equal); data curation (equal); formal analysis (supporting); investigation (equal); writing – original draft (equal).

## FUNDING INFORMATION

The bat surveys carried out from 1999 to 2020 were funded by Regione Autonoma Valle d'Aosta, through the Regional Museum of Natural Sciences of Saint‐Pierre and *Servizio Aree protette dell'Assessorato Agricoltura e Risorse naturali* (currently *Struttura Biodiversità, sostenibilità e aree naturali protette dell'Assessorato Ambiente, Trasporti e Mobilità sostenibile*). SM and LG received fundings from Biodiversa+, the European Biodiversity Partnership under the 2021–2022 BiodivProtect joint call for research proposals (Project "DarCo – the vertical dimension of conservation: A cost‐effective plan to incorporate subterranean ecosystems in post‐2020 biodiversity and climate change agendas), co‐funded by the European Commission (GA N°101052342) and with the funding organisations Ministry of Universities and Research (Italy), Agencia Estatal de Investigación – Fundación Biodiversidad (Spain), Fundo Regional para a Ciência e Tecnologia (Portugal), Suomen Akatemia – Ministry of the Environment (Finland), Belgian Science Policy Office (Belgium), Agence Nationale de la Recherche (France), Deutsche Forschungsgemeinschaft e.V. (Germany), Schweizerischer Nationalfonds (Grant N° 31BD30_209583, Switzerland), Fonds zur Förderung der Wissenschaftlichen Forschung (Austria), Ministry of Higher Education, Science and Innovation (Slovenia), and the Executive Agency for Higher Education, Research, Development and Innovation Funding (Romania). Additional support to SM and LG is provided by the P.R.I.N. 2022 “DEEP CHANGE” (2022MJSYF8) and “BATS‐SIGNALS” (20224MZ9HN) funded by the Ministry of Universities and Research (Italy). The authors acknowledge the support of NBFC to CNR, funded by the Italian Ministry of University and Research, P.N.R.R., Missione 4 Componente 2, “Dalla ricerca all'impresa”, Investimento 1.4, Project CN00000033.

## CONFLICT OF INTEREST STATEMENT

Authors declare no conflict of interest.

## Supporting information


Data S1.

## Data Availability

The authors confirm that the data supporting the findings of this study are available within the article and in Data S1.
